# Noninvasive Quantification of [^18^F]SynVesT-2 PET in Healthy Human Brains Using Simplified Reference Tissue Models

**DOI:** 10.2967/jnumed.125.271207

**Published:** 2026-05

**Authors:** Nira Hernández-Martín, Tutukhanim Balayeva, Mika Naganawa, Ruth H. Asch, Yiyun Huang, Richard E. Carson, Jean-Dominique Gallezot, Zhengxin Cai

**Affiliations:** 1 Department of Radiology and Biomedical Imaging, Yale School of Medicine, New Haven, Connecticut;; 2 Department of Psychiatry, Yale School of Medicine, New Haven, Connecticut;; 3 Department of Biomedical Engineering, Yale School of Medicine, New Haven, Connecticut; and; 4 Department of Pharmacology, Yale School of Medicine, New Haven, Connecticut

**Keywords:** radiotracer tissue kinetics, kinetic modeling, simplified reference tissue model, SRTM, synaptic vesicle glycoprotein 2A, [^18^F]SynVesT-2

## Abstract

PET imaging of synaptic vesicle glycoprotein 2A (SV2A) has proven to be a powerful research tool for neurologic disorders. Dynamic SV2A PET scans provide data related to cerebral blood flow and SV2A density, which have been shown to be altered in neurologic disorders such as Alzheimer disease. [^18^F]SynVesT-2, an SV2A PET tracer, has demonstrated fast brain kinetics and high specific binding in human brains. To improve clinical feasibility, we evaluated the performance of 3 simplified reference tissue models (SRTMs) in the quantification of [^18^F]SynVesT-2 PET data and the minimum scan times required for reliable estimation of relative cerebral blood flow and SV2A density. **Methods:** Data were pooled from 14 [^18^F]SynVesT-2 scans acquired from 9 healthy volunteers. An SRTM, SRTM with a fitted regionally coupled *k′*_2_ (SRTMC), and SRTM with a population-based *k′*_2_ (SRTM2) with the centrum semiovale and cerebellum as reference regions were used to calculate nondisplaceable binding potential (BP_ND_) and distribution volume ratio (DVR), respectively, as well as the relative tracer delivery rate (*R*_1_). Test–retest variability (TRV), absolute TRV, and the minimum scan duration for the reliable estimation of *R*_1_, BP_ND_, and DVR were additionally evaluated. **Results:** Despite time–activity curves being well-described by all 3 models, SRTM generated unreliable BP_ND_ and DVR values in 9% and 12% of the regions of interest, respectively. SRTMC and SRTM2 resulted in BP_ND_ and DVR values consistent with those generated from the 1-tissue compartment model. On the basis of the time stability analysis, BP_ND_ and DVR estimated using SRTM2 converged after 40 min. Using SRTM2, the TRV and absolute TRV estimated from 40-min dynamic scans were −1.0 ± 11.5% and 9.9 ± 5.8% for BP_ND_ and 1.7 ± 4.0% and 3.6 ± 2.5% for DVR. **Conclusion:** The parameters of relative cerebral blood flow (*R*_1_) and specific binding (BP_ND_ and DVR) can be reliably estimated from a 40-min dynamic [^18^F]SynVesT-2 PET scan by SRTM2, which is 30 min shorter than that required for [^11^C]UCB-J and [^18^F]SynVesT-1. The shortened scan time enables the clinical application of dynamic SV2A PET scans to maximize the physiologically relevant information attainable from a single scan.

## Visual Abstract

**Figure:**
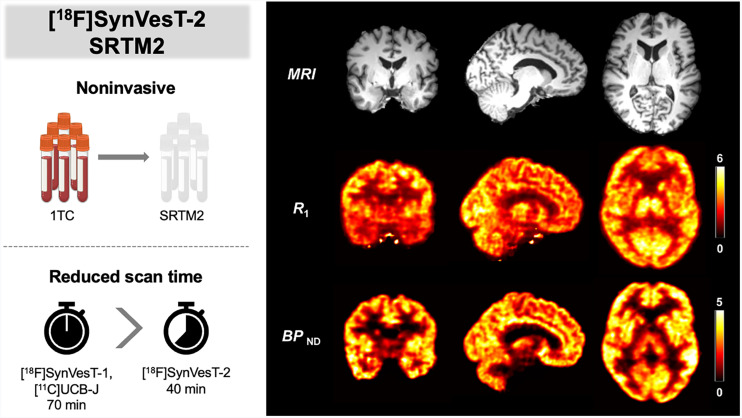

Synapses are the primary points of communication among billions of neurons in the mammalian central nervous system and serve as the elemental units of information processing (1). The number and configuration of synapses may change throughout life; however, synaptic dysfunction in specific brain regions could be an early biomarker of various neurodegenerative disorders and psychiatric diseases (1–3). The synaptic vesicle glycoprotein 2A (SV2A) is ubiquitously expressed in glutamatergic and γ-aminobutyric acid presynaptic neurotransmitter vesicles (4) and is an accepted biomarker of synaptic density (5). Further, PET with radiotracers targeting SV2A provided the first method for quantifying synaptic density in the living human brain (5,6). The noninvasive quantification of synaptic density has shown potential in the early detection, staging, and prognosis of a wide range of neurologic diseases (7–9).

Among the first-generation SV2A radiotracers, [^18^F]UCB-H (10) and [^11^C]UCB-A (11) have exhibited low specific signals and slow kinetics, respectively, whereas [^11^C]UCB-J has demonstrated fast kinetics and high specific-binding signals in the brain (5,12,13). An ^18^F-labeled radiotracer, [^18^F]SynVesT-1, developed by our team and others independently (14,15), showed similar brain uptake and kinetics with a high level of specific binding compared with [^11^C]UCB-J (12,16). We reported on an alternative SV2A ligand, [^18^F]SynVesT-2, which exhibited faster brain kinetics and required only a 30-min dynamic scan for the reliable estimation of parameters related to cerebral perfusion (*K*_1_) and SV2A-specific binding, as reflected by the nondisplaceable binding potential (BP_ND_) (8,17,18).

The gold standard for analyzing SV2A brain PET data involves the use of a metabolite-corrected arterial input function (AIF) (16,17,19). Although arterial cannulation is generally considered a safe procedure, it requires expertise not available at every PET clinic and may not be well-tolerated or suitable for certain populations. Consequently, simplified reference tissue models (SRTMs) have been tested as a noninvasive method for quantifying SV2A in the brain (13,20–22). This study aimed to investigate the performance of 3 SRTMs in the analysis of [^18^F]SynVesT-2 PET data, with the goal of eliminating the necessity for arterial blood collection and analysis. Additionally, we aimed to evaluate the minimum scan durations required to obtain stable outcome measures using these SRTMs.

## MATERIALS AND METHODS

Participants and study design; PET, MRI, and AIF measures; image registration details; and regions of interest are described in the supplemental material, available at http://jnm.snmjournals.org (23–25).

### Quantitative Analysis

Kinetic analysis was performed with regional time–activity curves. The 1-tissue compartment (1TC) model, using the metabolite-corrected AIF, was previously used to analyze this dataset (90-min scan data) (17). Here, we used 3 reference tissue models: (1) SRTM, (2) SRTM using a coupled fit approach for *k*′_2_ (clearance rate from the reference region) (SRTMC), and (3) SRTM with a population-based fixed value for *k*′_2_ (SRTM2) (13). *R*_1_, *k*_2_, and *k*′_2_ were estimated for each brain region (26). For the SRTMC, *k*′_2_ was estimated by jointly fitting all region-of-interest time–activity curves simultaneously and sharing the *k*′_2_ parameter across all regions of interest (27). Finally, for the SRTM2, a population average of 1TC *k*_2_ values for the reference region was used as the common *k*′_2_ for all brain regions and participants (28). The centrum semiovale (CS) was selected as the reference region to calculate the BP_ND_, as there is minimal expression of SV2A in the CS (17). The cerebellum was selected as a pseudoreference region to calculate DVR, which provided a more robust readout than BP_ND_ in a study of patients with mild cognitive impairment and Alzheimer disease using [^11^C]UCB-J (7). The relative performance for each reference tissue model fitting was evaluated using the Akaike Information Criterion and compared with 1TC model fitting.

The time stability of BP_ND_, DVR, and *R*_1_ was evaluated by fitting the regional time–activity curves with truncated acquisition times, from 10 to 80 min in 10-min increments. The percentage difference was computed with respect to the 90-min estimates. The acceptable minimum acquisition time for each brain region was determined by a mean percentage difference over all participants below 5% and intersubject SD (SD, understood as variability of that specific outcome measure for the 9 baseline scans in 9 healthy volunteers) below 10%.

## RESULTS

### SRTM Analysis

**FIGURE 1.**
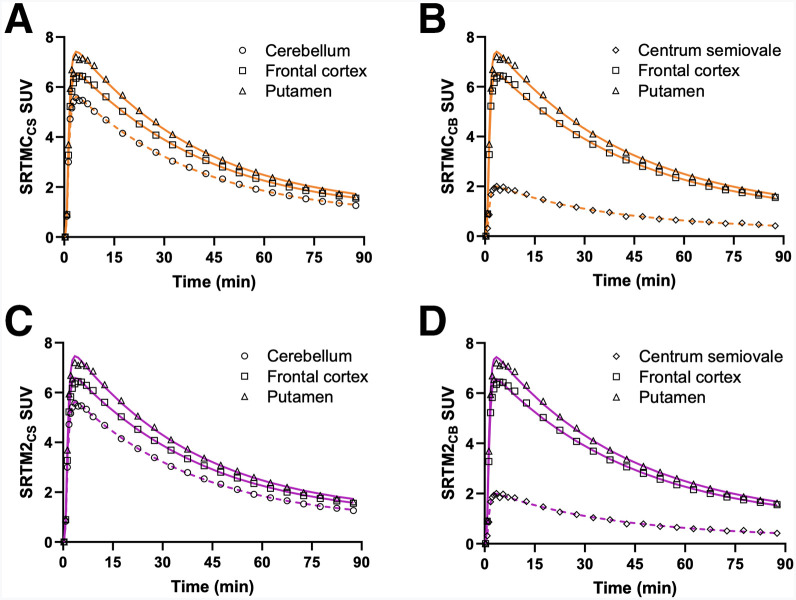
SUV time–activity curves of putamen, frontal cortex, and cerebellum or CS, using SRTMC (A and B) or SRTM2 (C and D). CB = cerebellum.

The fitted time–activity curves of representative brain regions (frontal cortex, putamen, CS, and cerebellum) demonstrated an excellent fit using the 3 models with both reference regions (Fig. 1; Supplemental Fig. 1). Comparable Akaike Information Criterion values were found for SRTM and SRTM2, with 64% of fittings for BP_ND_ and 84% of the fittings for DVR showing higher Akaike Information Criterion values for SRTM2 than SRTM (Supplemental Table 1). However, 26 SRTM DVRs and 20 SRTM BP_ND_ values yielded a total of 210 fits with relative SEs (rSEs) greater than 25%. In contrast, all SRTMC and SRTM2 BP_ND_ and DVR values were estimated reliably, with rSEs of less than 25%. The mean 1TC *k*_2_ of the reference regions (CS, 0.059 min^−1^; cerebellum, 0.052 min^−1^) was used as the population *k*′_2_ in the SRTM2 analysis. The *k*_2_ values obtained from the 1TC, SRTMC, and SRTM2 for each region, as well as the mean SRTMC *k*′_2_, are provided in Supplemental Table 2.

**FIGURE 2.**
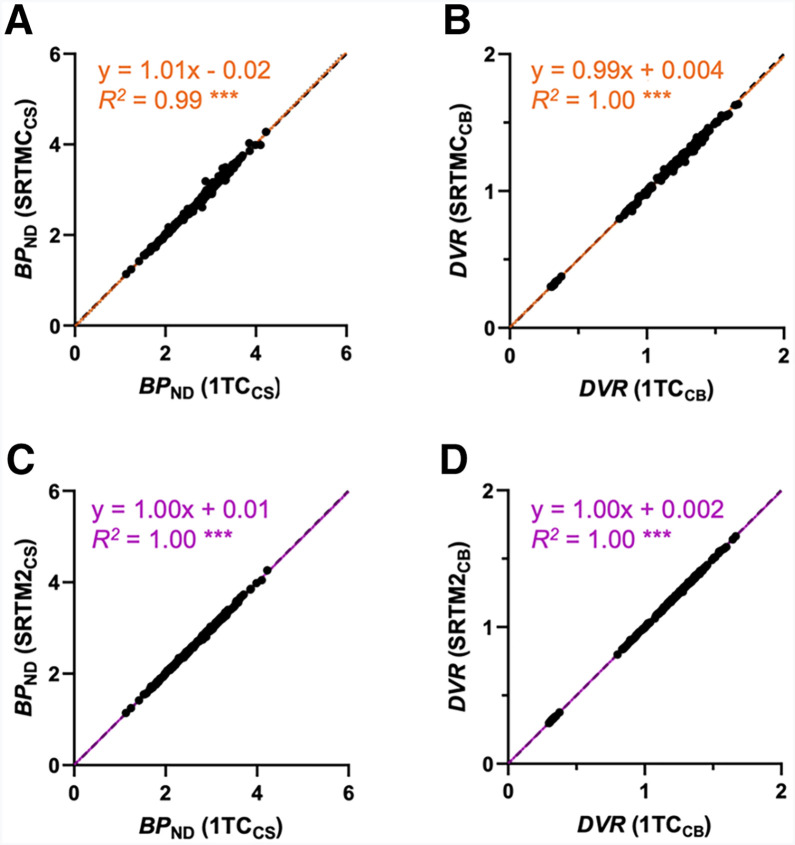
Correlation between outcome measures calculated with 1TC model, SRTMC (A and B), and SRTM2 (C and D) for BP_ND_ and DVR using 90-min dataset. All regions of interest are represented from 14 baseline scans of 9 participants. Black dashed line indicates line of identity; colored lines indicate linear regression. Data with rSE > 25% were excluded in plots shown in A and D. ***P < 0.001; CB = cerebellum.

To validate the results derived from SRTM, SRTMC, and SRTM2 analysis, we compared the BP_ND_ and DVR values generated using the 3 reference tissue models with those estimated from the 1TC model. After the outliers from the SRTM results were removed, a linear correlation was observed for SRTM (CS as reference, slope = 0.99, *R*^2^ = 0.99; cerebellum as reference, slope = 0.99, *R*^2^ = 1.00; Supplemental Figs. 1C and 1D), SRTMC (CS as reference, slope = 1.01, *R*^2^ = 0.99; cerebellum as reference, slope = 0.99, *R*^2^ = 1.00), and SRTM2 (CS as reference, slope = 1.00, *R*^2^ = 1.00; cerebellum as reference, slope = 1.00, *R*^2^ = 1.00; Fig. 2). To test the consistency of the outcome measures generated from the reference tissue models with those from the 1TC model, we calculated the percentage differences in BP_ND_ and DVR between the reference tissue models and the 1TC model (Tables 1 and 2). Even after the outliers were removed, SRTM exhibited higher variability from the 1TC model results (BP_ND_ = 0.4% ± 2.4%; DVR = −1.4% ± 1.6%), than SRTMC (BP_ND_ = 0.2% ± 1.7%; DVR = −0.6% ± 1.1%) and SRTM2 (BP_ND_ = −0.1% ± 0.8%; DVR = −0.1% ± 0.4%). Therefore, SRTM was not included in the subsequent analyses.

**TABLE 1.** Percentage Difference Between BP_ND_ Values Estimated Using SRTMs and 1TC Model*

		% Difference		
Region	1TC model	SRTM	SRTMC	SRTM2
Amygdala	2.6 ± 0.3	0.4 ± 3.6	0.2 ± 4.1	−0.4 ± 1.4
Anterior insula	3.0 ± 0.4	−0.4 ± 1.6	0.1 ± 1.7	−0.2 ± 0.7
Caudate	2.0 ± 0.4	−0.8 ± 0.8	0.2 ± 1.1	−0.1 ± 0.6
Cerebellum	2.0 ± 0.2	4.1 ± 3.3	0.3 ± 0.9	0.2 ± 0.8
DLPFC	3.0 ± 0.4	−0.1 ± 1.0	0.0 ± 0.8	0.0 ± 0.6
Frontal	2.6 ± 0.3	0.2 ± 1.1	0.1 ± 0.8	0.0 ± 0.6
Fusiform	2.9 ± 0.3	0.7 ± 2.9	0.2 ± 2.6	−0.3 ± 0.9
Hippocampus	1.8 ± 0.2	1.6 ± 2.4	0.2 ± 2.0	−0.2 ± 0.8
Occipital	2.7 ± 0.4	0.8 ± 2.2	0.1 ± 1.5	−0.1 ± 0.6
Orbitofrontal	2.7 ± 0.3	0.2 ± 1.1	0.1 ± 1.0	0.0 ± 0.6
Parietal	2.7 ± 0.4	−0.1 ± 1.3	0.1 ± 1.2	−0.1 ± 0.6
Putamen	3.1 ± 0.3	−0.7 ± 0.8	0.2 ± 1.0	−0.1 ± 0.5
Temporal	3.0 ± 0.4	0.5 ± 3.5	0.1 ± 2.0	−0.2 ± 0.8
Thalamus	2.0 ± 0.2	0.1 ± 2.5	0.3 ± 1.3	0.3 ± 1.1
Ventral striatum	3.6 ± 0.4	−0.6 ± 1.5	0.2 ± 1.7	−0.2 ± 0.7
All regions	2.5 ± 0.9	0.4 ± 2.4	0.2 ± 1.7	−0.1 ± 0.8

* CS was used as reference region.

DLPFC = dorsolateral prefrontal cortex.

Data are expressed as mean ± SD.

**TABLE 2.** Percentage Differences Between DVRs Estimated Using SRTMs and 1TC Model*

		% Difference		
Region	1TC model	SRTM	SRTMC	SRTM2
Amygdala	0.2 ± 0.1	−1.5 ± 1.4	−1.2 ± 2.4	−0.3 ± 0.6
Anterior insula	0.3 ± 0.1	−1.1 ± 0.8	−0.7 ± 0.9	−0.1 ± 0.3
Caudate	0.0 ± 0.1	−1.6 ± 1.8	−0.6 ± 0.6	−0.1 ± 0.3
CS	−0.7 ± 0.0	−2.0 ± 2.3	−0.5 ± 1.2	0.0 ± 0.4
DLPFC	0.3 ± 0.1	−2.2 ± 2.8	−0.5 ± 0.7	0.0 ± 0.3
Frontal	0.2 ± 0.1	−1.8 ± 2.4	−0.5 ± 0.7	0.0 ± 0.3
Fusiform	0.3 ± 0.1	−0.8 ± 2.1	−0.8 ± 1.6	−0.2 ± 0.4
Hippocampus	−0.1 ± 0.1	−0.6 ± 0.7	−0.7 ± 0.9	−0.1 ± 0.3
Occipital	0.2 ± 0.1	−0.9 ± 1.0	−0.6 ± 0.7	−0.1 ± 0.3
Orbitofrontal	0.2 ± 0.1	−0.7 ± 0.6	−0.5 ± 0.5	0.0 ± 0.2
Parietal	0.2 ± 0.1	−1.0 ± 0.9	−0.6 ± 0.6	−0.1 ± 0.2
Putamen	0.3 ± 0.1	−1.4 ± 1.4	−0.6 ± 0.6	−0.1 ± 0.3
Temporal	0.3 ± 0.1	−1.4 ± 0.7	−0.8 ± 1.0	−0.2 ± 0.3
Thalamus	0.0 ± 0.1	−1.7 ± 1.5	−0.2 ± 1.8	0.2 ± 0.5
Ventral striatum	0.5 ± 0.1	−1.5 ± 0.8	−0.7 ± 1.0	−0.2 ± 0.3
All regions	0.1 ± 0.3	−1.4 ± 1.6	−0.6 ± 1.1	−0.1 ± 0.4

* Cerebellum was used as reference region.

DLPFC = dorsolateral prefrontal cortex.

Data are expressed as mean ± SD.

**FIGURE 3.**
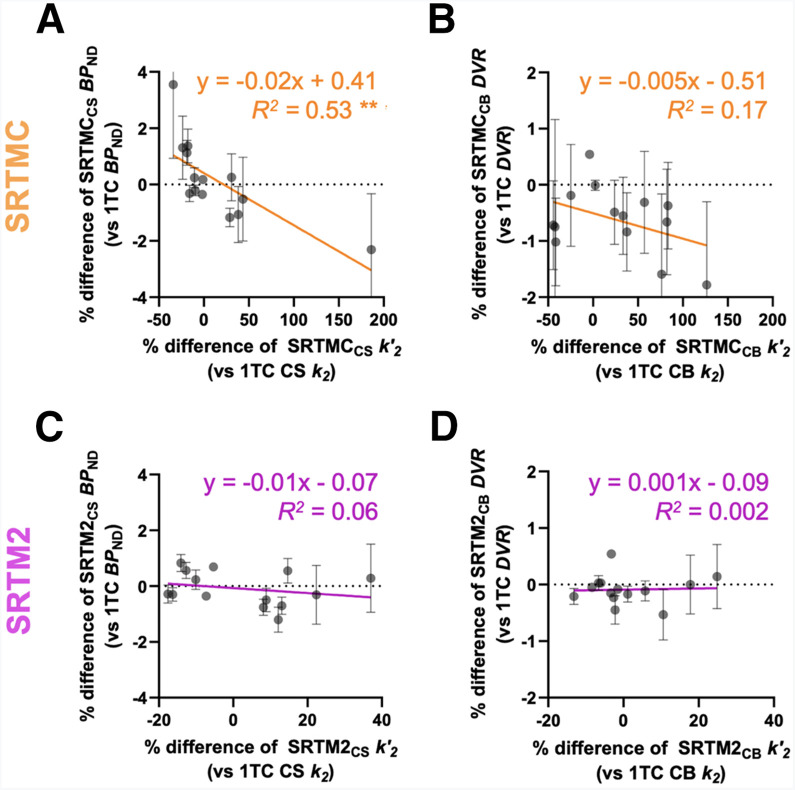
Scatter plots comparing percentage differences between SRTMC/SRTM2 and 1TC model estimates of BP_ND_ and DVR, with percentage differences between SRTMC/SRTM2 *k*′_2_ and 1TC model *k*_2_ values for reference region from 14 scans of 9 participants. Data points and error bars are mean and SD across all selected regions of interest in each scan. Note that SRTMC and SRTM2 have different scales for their axes. CB = cerebellum. **P < 0.001.

To test if the bias in *k*′_2_ had any impact on the bias of the outcome measures, we conducted a correlational analysis of the BP_ND_ and DVR bias relative to the bias in *k*′_2_ (Fig. 3). The SRTMC showed an overestimation of BP_ND_ of up to 3.6% and an underestimation of up to 2.3% and was negatively correlated with the bias of *k*′_2_ (slope = −0.02, *R*^2^ = 0.53, *P* = 0.003) (Fig. 3A). However, SRTM2 demonstrated an accurate estimation of BP_ND_, exhibiting an average bias of 1.2%, independent of the bias in *k*′_2_ (slope = −0.01, *R*^2^ = 0.06, *P* = 0.41) (Fig. 3C). Further, both SRTMC and SRTM2 led to accurate estimates of DVR that were independent of the bias in *k*′_2_, with higher variability in the SRTMC DVR relative to the SRTM2 DVR (Figs. 3B and 3D).

The aforementioned relationship between the bias in *k*′_2_ and the bias in BP_ND_ and DVR can be explained by the strong linear correlation between the bias in *k*_2_ across regions and the bias in *k*′_2_ for SRTMC and SRTM2 (Supplemental Fig. 2).

### Time Stability Analysis

**FIGURE 4.**
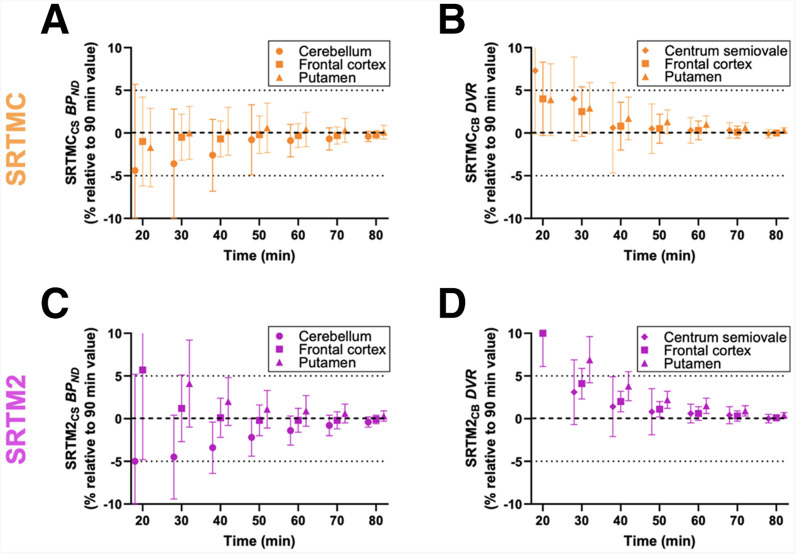
Time stability of BP_ND_ and DVR estimated using SRTMC (A and B) and SRTM2 (C and D). Percentage differences are presented for estimated outcome measures from truncated scan durations compared with those from 90-min scan data. Data are mean ± SD per region of interest (cerebellum, frontal cortex, and putamen) for 9 baseline scans in 9 participants. In panel A, data with an rSE of greater than 25% were excluded. CB = cerebellum.

Time stability was investigated to determine the minimum scan time required for the reliable and accurate estimation of *R*_1_, BP_ND_, and DVR using SRTMC and SRTM2. There were 15 SRTMC BP_ND_ values with rSEs greater than 25% at 20, 30 and 40 min, from a total of 210 fits per time point, which were excluded from the analysis. The SRTMC BP_ND_ converged at 50 min after injection (cerebellum, −0.8% ± 4.1%; frontal cortex, −0.2% ± 2.2%; putamen, 0.6 ± 2.9%) (Fig. 4A), and the SRTMC DVR converged at 40 min after injection (CS, 0.6% ± 5.3%; frontal cortex, 0.8% ± 2.8%; putamen, 1.7 ± 2.5%) (Fig. 4B). Notably, the SRTM2 BP_ND_ (cerebellum, −3.4% ± 3%; frontal cortex: 0.1% ± 2.3%; putamen, 2.0% ± 2.8%) and DVR (cerebellum, 1.4% ± 3.5%; frontal cortex, 2.0% ± 1.2%; putamen, 3.8% ± 1.7%) converged at 40 min after injection (Figs. 4C and 4D). The minimum scan time using SRTMC and SRTM2 for each brain region is provided in Supplemental Figure 1 and Supplemental Table 3.

### Test–Retest Reproducibility

The test–retest variability (TRV) and absolute TRV (aTRV) for the SRTMC and SRTM2 were first calculated for BP_ND_ (Table 3) and DVR using the full 90-min dataset (Table 4). The TRV was low for both the SRTMC (BP_ND_, 0.3% ± 12.8%; DVR, 1.6% ± 3.9%) and SRTM2 (BP_ND_, −0.1% ± 12.3%; DVR, 1.9 ± 3.8%). Both models exhibited a lower aTRV for DVR (SRTMC, 3.5% ± 2.2%; SRTM2, 3.6% ± 2.2%) than BP_ND_ (SRTMC, 11.6% ± 5.3%; SRTM2, 11.0% ± 5.3%). The TRV and aTRV were also calculated for BP_ND_ and DVR using their minimum scan durations (Supplemental Tables 4 and 5). Finally, TRV and aTRV were evaluated for *R*_1_ (Supplemental Tables 6 and 7).

**TABLE 3.** TRV and aTRV of BP_ND_ Values from SRTMC and SRTM2 Analyses of 90-Minute Dataset*

	TRV (%)		aTRV (%)	
Region	SRTMC	SRTM2	SRTMC	SRTM2
Amygdala	2.0 ± 13.4	0.7 ± 11.5	11.3 ± 5.2	9.0 ± 5.6
Anterior insula	1.0 ± 13.2	0.4 ± 12.4	11.2 ± 4.4	10.2 ± 4.7
Caudate	−2.5 ± 16.3	−2.9 ± 16.2	13.8 ± 6.0	13.7 ± 6.0
Cerebellum	−2.5 ± 11.8	−2.8 ± 11.4	9.7 ± 5.3	9.7 ± 4.7
DLPFC	−1.5 ± 11.2	−1.7 ± 10.9	9.3 ± 4.6	9.0 ± 4.7
Frontal	−0.7 ± 12.6	−0.9 ± 12.2	10.6 ± 4.4	10.2 ± 4.6
Fusiform	3.9 ± 15.2	3.0 ± 13.9	13.8 ± 3.9	12.2 ± 4.3
Hippocampus	3.8 ± 16.7	3.1 ± 16.0	13.6 ± 8.1	12.4 ± 8.6
Occipital	1.0 ± 14.4	0.6 ± 13.8	11.8 ± 5.8	11.2 ± 6.0
Orbitofrontal	1.1 ± 14.1	0.8 ± 13.6	12.4 ± 2.8	11.9 ± 3.0
Parietal	−0.1 ± 16.1	−0.5 ± 15.7	12.7 ± 7.6	12.1 ± 7.9
Putamen	−1.1 ± 11.2	−1.3 ± 11.0	9.3 ± 4.4	9.3 ± 3.8
Temporal	1.1 ± 14.3	0.5 ± 13.4	12.3 ± 4.2	11.2 ± 4.9
Thalamus	−1.3 ± 16.8	−0.9 ± 17.1	13.4 ± 7.9	13.4 ± 8.2
Ventral striatum	0.3 ± 12.0	−0.4 ± 11.3	9.3 ± 5.8	9.3 ± 4.6
All regions	0.3 ± 12.8	−0.1 ± 12.3	11.6 ± 5.3	11.0 ± 5.3

* CS was used as reference region.

DLPFC = dorsolateral prefrontal cortex.

Data are expressed as mean ± SD.

**TABLE 4.** TRV and aTRV of DVRs from SRTMC and SRTM2 Analyses of 90-Minute Dataset*

	TRV (%)		aTRV (%)	
Region	SRTMC	SRTM2	SRTMC	SRTM2
Amygdala	2.6 ± 2.1	2.2 ± 2.3	2.6 ± 2.1	2.4 ± 2.0
Anterior insula	2.0 ± 3.2	2.1 ± 3.2	3.4 ± 1.0	3.4 ± 1.4
Caudate	−0.6 ± 4.5	−0.3 ± 4.1	3.3 ± 2.7	3.0 ± 2.3
CS	1.5 ± 7.3	2.0 ± 7.5	5.9 ± 3.6	6.5 ± 2.9
DLPFC	0.3 ± 3.4	0.7 ± 3.7	2.8 ± 1.3	3.0 ± 1.7
Frontal	0.8 ± 2.9	1.3 ± 3.2	2.3 ± 1.7	2.5 ± 2.0
Fusiform	4.2 ± 4.5	3.9 ± 4.0	5.3 ± 2.6	5.0 ± 2.1
Hippocampus	3.6 ± 2.6	3.7 ± 2.4	3.6 ± 2.6	3.7 ± 2.4
Occipital	2.0 ± 3.7	2.3 ± 3.6	3.4 ± 2.0	3.5 ± 2.1
Orbitofrontal	2.1 ± 4.4	2.5 ± 4.7	4.0 ± 2.1	4.3 ± 2.6
Parietal	1.1 ± 4.1	1.4 ± 4.1	3.5 ± 1.9	3.6 ± 1.9
Putamen	0.6 ± 2.3	0.9 ± 2.3	1.7 ± 1.5	1.9 ± 1.3
Temporal	2.1 ± 4.3	2.1 ± 4.0	4.2 ± 1.5	4.0 ± 1.3
Thalamus	0.5 ± 4.4	1.4 ± 4.3	3.4 ± 2.3	3.0 ± 3.1
Ventral striatum	1.6 ± 4.4	1.5 ± 4.3	3.9 ± 1.9	3.6 ± 2.3
All regions	1.6 ± 3.9	1.9 ± 3.8	3.5 ± 2.2	3.6 ± 2.2

* Cerebellum was used as reference region.

DLPFC = dorsolateral prefrontal cortex.

Data are expressed as mean ± SD.

## DISCUSSION

Maintaining synapse homeostasis and delaying synapse loss are potential objective surrogate endpoints in the clinical evaluation of new disease-modifying treatments for neurodegenerative diseases. Therefore, as an in vivo imaging and quantification tool for synaptic density, SV2A PET has been broadly used in clinical studies of neurodegenerative diseases and psychiatric disorders. Although the SUV ratio calculated from static SV2A PET scans at a predetermined imaging window provides information on relative synaptic density, dynamic PET scans provide important parameters related to cerebral blood flow (29). These parameters have been used to measure the extent of hypoperfusion in neurodegenerative diseases using PET tracers targeting β-amyloid plaque (30–32) and tau tangles (33) and have been shown to be clinically relevant in patients with Alzheimer disease (34). In this study, we explored 3 reference region–based quantification methods for [^18^F]SynVesT-2 to obtain quantitative measures of both relative cerebral blood flow and synaptic density. We found that both SRTMC and SRTM2 provided reliable *R*_1_, BP_ND_, and DVR estimations that were consistent with those derived from a 1TC model. Furthermore, [^18^F]SynVesT-2 requires only a 40-min dynamic scan to reliably and reproducibly estimate *R*_1_, BP_ND_, and DVR values using the SRTM2.

In contrast to 1TC kinetic modeling, reference region–based analysis provides a noninvasive approach for PET quantification without the need of AIF. In SV2A PET imaging studies, the CS has been explored as a reference region to calculate BP_ND_, as the CS has lower radiotracer uptake when compared with gray matter regions and a minimal blocking effect (17,35). However, the CS is not a perfect reference region, as [^18^F]SynVesT-2 demonstrated 20% specific binding in the CS, which is similar to the 20% and 22% specific-binding signals observed for [^11^C]UCB-J and [^18^F]SynVesT-1, respectively (17,35,36). Alternatively, we estimated DVR using the cerebellum as a pseudoreference region, as it demonstrated lower intersubject variability than did BP_ND_ in a previous SV2A PET imaging study of Alzheimer disease (7). Thus, the cerebellum could be a suitable reference region for cross-sectional studies, as long as the total binding in the cerebellum is unaffected by the disease.

In this study, the SRTM, SRTMC, and SRTM2 fit well with the brain time–activity curves. In addition to the SRTM, multilinear reference tissue methods have also been proposed (37). These methods produce estimates similar to those of the SRTM and also permit fixing the value of *k*′_2_ to reduce noise. The SRTM was unable to provide reliable estimation of BP_ND_ or DVR values for many fittings and exhibited a greater degree of bias for the rest when compared with the 1TC model results. Therefore, we focused on the SRTMC and SRTM2 in our analysis, both of which have fewer independent parameters than the SRTM, with the caveats that the SRTMC *k*′_2_ estimates are dependent on the brain regions selected in the analysis, and the SRTM2 *k*′_2_ may be affected by certain disease states. Both models yielded BP_ND_ and DVR values with a high degree of correlation with 1TC model results. Furthermore, the outcome measures from the SRTMC and SRTM2 are highly consistent with those from the 1TC model, with mean absolute percentage differences of less than 1% for BP_ND_ and DVR. Compared with the 1TC model, the biases of *k*′_2_ and *k*_2_ correlated well with each other in the SRTMC and SRTM2, which explains why the bias in BP_ND_ and DVR (calculated using the ratio of *k*′_2_ and *k*_2_*)* were insensitive to the bias in *k*′_2_. Both SRTMC and SRTM2 demonstrated high reproducibility, with a low aTRV using the CS or cerebellum as the reference region on the 90-min dataset and a minimum scan time.

Previously investigated SV2A PET tracers, [^11^C]UCB-J and [^18^F]SynVesT-1, require a minimum scan duration of 1 h for reliable estimation of kinetic parameters, making the implementation of dynamic scan protocols at imaging clinics a bit challenging for patients. [^18^F]SynVesT-2 exhibited faster kinetics, while maintaining high specific-binding signals, requiring only a 30-min dynamic scan for the reliable estimation of *K*_1_ and BP_ND_ using the 1TC model (17). As a noninvasive alternative, SRTM2 analysis required a minimum scan time of 40 min to obtain stable BP_ND_ and DVR estimates for [^18^F]SynVesT-2, which is 30 min shorter than the scans required by [^11^C]UCB-J and [^18^F]SynVesT-1. However, we must establish whether a 40-min scan provides sufficient data for a voxel-based SRTM2, which we plan to address in future work.

Caution is needed regarding the selection of the *k*′_2_ and reference region. The selection of the reference region may vary, depending on the specific pathology under investigation, and the population *k*′_2_ must therefore be calibrated for each specific setting. For example, in patients with mild cognitive impairment and Alzheimer disease, the synapse density of the cerebellum is unchanged compared with healthy controls, making the cerebellum a reasonable reference region for group comparisons. Therefore, it is strongly preferable to perform an initial 1TC analysis in the population of interest, which requires AIF. When this is not feasible because of logistic limitations, the SRTMC represents an alternative analytic approach.

## CONCLUSION

Both the SRTMC and SRTM2 can be used in the quantitative analysis of human brain [^18^F]SynVesT-2 PET data to provide estimates of relative cerebral blood flow and synaptic density, without the need for arterial blood collection. With the use of a population-based *k*′_2_, SRTM2 analysis could reduce the dynamic scan time to 40 min, which is 30 min shorter than that required by [^11^C]UCB-J and [^18^F]SynVesT-1.

## DISCLOSURE

This research was supported by grants R01AG052560, R01AG065474, R01AG058773, and R01NS123183 from the National Institutes of Health and by research grants from the Michael J. Fox Foundation and the Archer Foundation. This work was also supported by CTSA grant UL1 RR024139 awarded jointly by the National Center for Research Resources and the National Center for Advancing Translational Sciences, components of the National Institutes of Health. The contents of this publication are solely the responsibility of the authors and do not necessarily represent the official view of the National Institutes of Health. The radioligands [^18^F]SynVesT-1/2 are covered by patent WO2018152339A1. No other potential conflict of interest relevant to this article was reported.
